# Effect of Material and Structure of Ultra-High-Molecular-Weight Polyethylene Body Armor on Ballistic Limit Velocity: Numerical Simulation

**DOI:** 10.3390/polym16212985

**Published:** 2024-10-24

**Authors:** Jiang Bian, Kaida Dai, Xiaojiang Lv, Zilu Huang, Guangrun Wu, Yuan Zhang

**Affiliations:** 1 State Key Laboratory of Explosion Science and Technology, Beijing Institute of Technology, Beijing 100081, China; 18681981207@163.com (J.B.); tuanzi00000@163.com (X.L.); 3120230124@bit.edu.cn (Z.H.); 3220230166@bit.edu.cn (G.W.); 2 Biomolecular Materials Science Research Center, Beijing 100120, China

**Keywords:** UHMWPE body armor, FSP, ballistic limit velocity, numerical simulation

## Abstract

The material properties and structural characteristics of ballistic composites are crucial to their ballistic performance. A numerical model of a 1.1 g FSP penetrating a UHMWPE target plate was established in this paper. The numerical results show that the failure process of the body armor target plate primarily involves shear failure, interlayer delamination, and tensile failure. Based on this, further research was conducted on the influence of material properties and structural characteristics on the ballistic limit velocity of the UHMWPE armor plate. Furthermore, the study evaluates the effects of elastic modulus, tensile strength, shear strength, number of layers, and interlayer strength on the ballistic limit velocity of UHMWPE body armor. The findings reveal that the ballistic limit velocity is most sensitive to changes in shear strength, with variation rates ranging from −18% to +11%, showing an approximate positive correlation, while the elastic modulus has the smallest impact on ballistic limit velocity, with variation rates ranging from −2% to +4%. Additionally, appropriate interlayer strength can improve the ballistic limit velocity of the body armor to a certain extent. This study provides theoretical methods and recommendations for optimizing anti-penetration performance of UHMWPE body armor.

## 1. Introduction

With advancements of material science technology, the soldier armor has undergone significant evolution from ancient armors to modern soft body armors. Soft body armors are composed of various high-strength fibers, which dissipate or distribute energy through high-performance fibers, continuously enveloping the bullet or fragment to mitigate its kinetic energy, thereby achieving ballistic protection.

In contemporary combat environments, fragments have become the primary cause of injuries to military personnel [1,2], motivating the study of the penetration resistance of soft body armors against fragments. Common fibers used in modern soft body armors include glass fibers [3,4,5,6], carbon fibers [7,8,9], aramid fibers [10,11,12,13,14], and Ultra-High-Molecular-Weight Polyethylene (UHMWPE) fibers [15,16,17]; the performance parameters of the above several common fibers are shown in Table 1 [18]. UHMWPE fibers, in particular, exhibit a higher breaking elongation rate and a specific strength that is greater than that of aramid fibers, alongside excellent energy absorption characteristic. At the same areal density, UHMWPE fiber composite materials offer approximately 25% greater bulletproof capability compared to aramid fiber composites [19]. Therefore, comprehending the penetration resistance mechanism of UHMWPE soft body armors is critical.

Zhang et al. [20] developed an analytical model to describe the behavior of UHMWPE cross-ply plates under ballistic impact, dividing the impact process into two stages—local failure and bulging deformation. They found that the maximum strain of the UHMWPE layer beneath the penetrating projectile is determined by the thickness of the non-penetrated laminate and the bulging protrusion. Additionally, the longitudinal wave speed and maximum tensile strain of the UHMWPE laminate determine its ballistic limit. He et al. [21] conducted an in-depth study on the ballistic response mechanism of ultra-high molecular weight polyethylene (UHMWPE) composites through experimental and simulation analyses. They revealed three fundamental response modes-localized response, structural response, and coupled response, under varying projectile velocities and laminate thicknesses. Based on the penetration resistance of projectiles, they proposed a new theoretical model to predict the ballistic performance of UHMWPE composites. The model demonstrated high predictive accuracy in experimental validation, providing significant theoretical support and guidance for the design and optimization of ballistic protection equipment. Haris and Tan [22] explored new configurations beyond traditional cross-laminated structures to enhance the ballistic protection performance of UHMWPE laminates. Their research found that a multi-layered system composed of several thin UHMWPE laminates simply stacked together demonstrates higher ballistic protection under impact compared to a single thick laminate, especially when there are no gaps between the laminates. Additionally, a laminated configuration formed by rotating adjacent laminates by 45° and bonding them together exhibits a higher ballistic limit in thin laminates compared to traditional cross-laminated structures, though the improvement is less significant in thicker laminates. These findings suggest that optimizing the stacking and configuration of laminates can significantly enhance the ballistic protection capabilities of UHMWPE materials.

Heisserer et al. [23] studied the ballistic penetration depth of Dyneema^®^ composites at different speeds to understand their protective performance under high-speed impacts. The experimental study with rigid steel spheres penetrating Dyneema^®^ HB26 composite laminates showed an almost linear relationship between penetration depth and impacting kinetic energy, suggesting a consistent penetration mechanism across the testing velocity range. Additionally, the energy absorbed per unit area increases with increasing impact velocity and decreasing plate thickness, up to 40 J/(kg/m²). Shen et al. [24], Xie et al. [25], and Tan et al. [14] examined the effects of different projectile head shapes on the deformation and energy dissipation mechanisms of UHMWPE composites by experiments and simulations. The results indicated that projectile head shape significantly affects the deformation and energy dissipation of UHMWPE composites, with sharp projectiles having the strongest penetration ability and flat projectiles the weakest. Sharp projectiles primarily penetrate through shear damage, causing local damage to areas, while blunt projectiles penetrate via a combination of shear plugging, tensile deformation, and large-area delamination. Hu et al. [26] investigated the ballistic performance of ultra-high-molecular-weight polyethylene (UHMWPE) fiber-reinforced laminates under different projectile shapes, layer spacing, and lamination angles through experiments and numerical simulations. The study found that flat projectiles had the weakest penetration ability, while increasing the layer spacing reduced the ballistic performance of the laminates. Additionally, a slight increase in the lamination angle could marginally improve ballistic performance, especially at lower impact velocities. The findings suggest that adjusting these parameters can optimize the ballistic protection of UHMWPE laminates, providing a scientific basis for designing more effective bullet-resistant materials.

Li et al. [27,28] investigated the ballistic performance of hybrid laminated composites made of carbon, Kevlar, and ultra-high-molecular-weight polyethylene (UHMWPE) fibers through experiments and finite element analysis. They found that the hybrid fiber materials exhibited better energy absorption and enhanced ballistic efficiency under impact, particularly in the UHMWPE/Kevlar hybrid structure where Kevlar fibers were used as the front layer. This configuration proved to be more effective in absorbing impact energy and increasing the ballistic limit. Moreover, by adjusting the fiber combination and the layering sequence, the ballistic performance of the composites can be optimized, making them more broadly applicable in military and protective fields. Zhang et al. [29] studied the effects of different constraint conditions on the ballistic performance of B4C/C/UHMWPE composite armor. They conducted ballistic experiments with 12.7 mm armor-piercing incendiary rounds using a target frame with adjustable constraint conditions, combined with numerical simulations to analyze the impact of the distance between the impact point and the constraint conditions on the armor’s performance. The results showed that the ballistic performance of the composite armor was significantly affected by the flexibility of its constraint conditions. Increasing the flexibility improved its protective effect, and further enhancement in ballistic performance could be achieved by optimizing structural design, such as reducing the number of bolts and avoiding projectile impact in bolt areas.

Researchers have conducted extensive studies on the ballistic performance of UHMWPE composite plates with varying thicknesses [30,31,32,33]. The findings show that increasing the thickness of the composite material significantly helps to enhance the ballistic properties. Thin UHMWPE plates primarily fail through fiber tension, displaying considerable deformation and bulging. As the plate thickness increases, a two-stage penetration process is observed, of which the initial stage involves shear plug formation, followed by the transition plane formation and rear plate bulging. Chen et al. [34] investigated the mechanical properties of UHMWPE fibers under different strain rates by conducting quasi-static and high-speed tensile tests. They examined the uniaxial tensile performance of UHMWPE fiber laminates within a strain rate range of 0.0013 to 163.78 s^−1^. The results showed that while the mechanical properties of UHMWPE fiber laminates are not sensitive to thickness, both strength and elastic modulus increase with increasing strain rate. The fracture modes of UHMWPE fiber laminates differ between low and high strain rates, which affects their tensile strength. Karthikeyan and Russell [35] explored the ballistic impact response of UHMWPE laminates at different areal densities and examined the effect of interlayer separation (pre-delamination) on bulletproof performance. By testing ballistic impact on UHMWPE laminates with varying areal densities and analyzing their failure modes and energy dissipation mechanisms, they determined the critical areal density. Below this density, the plate exhibits a binary failure mode (complete penetration or no damage), and above it, a progressive failure mode (partial fiber damage) is observed. Under the progressive failure mode, two different deformation zones are noted near the impact site and the far end, with the far end being 6.5 times more effective in dissipating projectile kinetic energy. Zhang et al. [36] investigated the effect of curvature on the backface deformation of ultra-high-molecular-weight polyethylene (UHMWPE) laminates under high-velocity impact. The results showed that an increase in curvature leads to a more concentrated deformation area, greater apex displacement, and a shift in the deformation mode from predominantly membrane stretching to a combination of membrane stretching and bending. Theoretical analysis revealed that the increase in curvature slows down the bending wave speed, reducing the material area involved in the deformation, which results in larger apex displacement. The study suggests that by selecting smaller curvatures and enhancing the bending stiffness of the laminates, backface deformation can be mitigated, leading to the design of UHMWPE laminates with improved ballistic performance. Lässig et al. [37] studied effects of UHMWPE material parameters on residual velocity and damage area, providing direction for future material improvements. Heisserer [38] analyzed the material parameters of UHMWPE, such as density, modulus, and material strength, to study their impact on ballistic performance. The results show that density has little effect on V50, and increasing the modulus at constant strength reduces performance, while increasing strength at a constant modulus can significantly improve performance. However, there is limited research that comprehensively considers the failure process of UHMWPE and quantifies the effects of its material parameters and structural characteristics on its ballistic limit velocity.

This paper systematically studies the effects of material parameters and structural characteristics on the ballistic limit velocity of body armor plates using numerical simulation. A finite element model was developed to simulate the penetration process of a 1.1 g fragment-simulating projectile (FSP) through a UHMWPE ballistic target, and its reliability was verified through experimental results. After analyzing the penetration mechanism of the target, the study also explored the influence of various parameters-including elastic modulus, tensile strength, shear strength, layer count, and interlayer strength-on the penetration resistance of UHMWPE body armor. Additionally, the influence pattern of these parameters on ballistic performance is analyzed in detail. This analysis clearly reveals which parameters have a greater impact on the ballistic limit velocity of UHMWPE. In the future optimization design of body armor, these more sensitive factors can be targeted to enhance the ballistic performance of the armor.

## 2. Numerical Model

### 2.1. Finite Element Model

Using the Hypermesh 2022 software, models of the body armor target plate, fragment, and gelatin were created and meshed. To save computational time, all models using a quarter model and symmetric constraints were set on the symmetric boundaries. The body armor target plate is 200 mm in length and width, with a total thickness of 4.124 mm.

The body armor target plate was modeled by using a layered approach, with 23 layers in total. To improve computational accuracy and save time, each layer was meshed by using a transitional meshing method, with the mesh refined in the area where the fragment contacts the body armor target plate. Table 2 shows the effect of mesh size on the numerical results. Taking both accuracy and computation time into consideration, the minimum size of the transition mesh was set to 0.4 mm.The body armor target plate has 451,950 elements and 913,560 nodes in total. The gelatin, 200 mm in length and width and 112 mm in thickness, was similarly meshed by using a transitional meshing method, with the smallest element size of the transitional mesh being 1 mm. The gelatin has 21,956 elements and 227,519 nodes in total. The fragment’s mesh size was controlled between 0.1 and 0.5 mm, with 936 elements and 1232 nodes in total. All the finite element models used hexahedral and eight-node solid elements.

**Figure 1:**
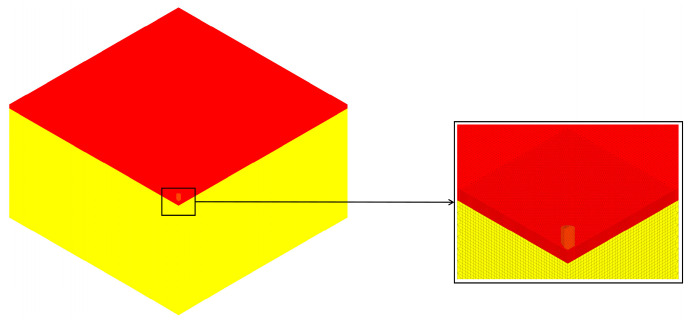
Finite element model.

Using the finite element software LS-DYNA 2022R1 for numerical simulation, the metric system was set to cm-g-μs. The contact between each layer of the body armor target plates and between the body armor target plates and the gelatin block was set to *CONTACT_AUTOMATIC_SURFACE_TO_SURFACE, allowing the model to automatically detect and establish contact relationships between the master and slave surfaces. The contact between the fragments and the target plates and gelatin was set to *CONTACT_ERODING_SURFACE_TO_SURFACE, a special contact algorithm used to simulate the erosion phenomenon during the contact process. The failure mode of the body armor target plates and the gelatin block was added using the MAT_ADD_EROSION keyword. In addition to the symmetric constraints on the symmetric boundaries, a fully fixed constraint was also applied to the back face of the gelatin block. The finite element model is shown in Figure 1.

### 2.2. Material Model

The material model for the body armor target was selected as MAT_COMPOSITE_FAILURE_SOLID_MODEL (MAT 59), based on elastoplastic theory. MAT59 is an orthotropic material model, which uses maximum stress failure criterion. These include in-plane tensile failure, through-thickness tensile and shear failure, longitudinal compressive failure, and through-thickness and transverse compressive failure. Once the maximum load is reached along a particular direction, the corresponding stiffness is set to zero in a time interval of 100 times steps [39,40]. The material parameters for the UHMWPE body armor target plate used in this paper were those of the UHMWPE laminate provided by Hu et al. [41], with some adjustments. The specific parameters are listed in Table 3.

The 1.1 g FSP is made of 45# steel, and its material model is described using a combination of the JOHNSON-COOK model and the GRUNEISEN equation of state. The material parameters are listed in Table 4 [42].

The gelatin material model is described using the material model MAT_ELASTIC_PLASTIC_HYDRO (MAT_10) along with the EOS_LINEAR_POLYNOMIAL equation of state. The material parameters are listed in Table 5 [43].

## 3. Validation and Analysis

### 3.1. Validation

The reliability of the finite element model established above was validated by cross-referencing with the ballistic limit velocity V_50_ obtained from the control experiment. The ballistic limit velocity V_50_ is the arithmetic mean of all projectile velocities when the projectile velocities are close and 50% of the projectiles penetrate the target plate, reflecting the ballistic limit velocity. This is an important indicator for evaluating the ballistic performance of materials through ballistic impact tests. The testing method for the ballistic limit velocity V_50_ of UHMWPE body armor target plates refers to the GB/T 32497-2016 [44]. The standard requires that when two projectiles effectively hit the target, the arithmetic mean of the velocities at the measurement points of these two projectiles is taken as the ballistic limit velocity V_50_ of the laminate (as shown in Formula (1)) if the velocity difference between the two bullets is not greater than 15 m·s^−1^ and one penetrates while the other is blocked.

(1) $$V_{50}=1/2(V_{\mathrm{penetration}}+V_{\mathrm{blocking}}),\;\mathrm{when}(V_{\mathrm{penetration}}-V_{\mathrm{blocking}})\leq 15,$$

According to the ballistic experiment, the ballistic limit velocity V_50_ of the 1.1 g FSP penetrating the UHMWPE body armor target plate is 565 m·s^−1^. The numerical simulation results are V_penetration_ = 560 m·s^−1^ and V_blocking_ = 550 m·s^−1^ and, thus, the ballistic limit velocity V_50_ obtained from the numerical simulation is 555 m·s^−1^. The results of the ballistic limit velocity V_50_ from the above experiment and numerical simulation are listed in Table 6.

From the results obtained in Table 6, it can be seen that the ballistic limit velocity V_50_ from both the experiment and the numerical simulation are close, with a discrepancy of only −1.8%. To reduce the errors between the experiment and numerical simulation, the experimental conditions (such as impact location and constraint method) can be closely replicated. As for environmental factors like temperature and humidity, since this paper primarily focuses on the effect of the parameters and structure of the UHMWPE ballistic target on its ballistic limit velocity, the errors caused by these environmental factors can be ignored. Therefore, the above finite element model meets the requirements for subsequent numerical calculations.

### 3.2. Ballistic Penetration Process Analysis

**Figure 2:**
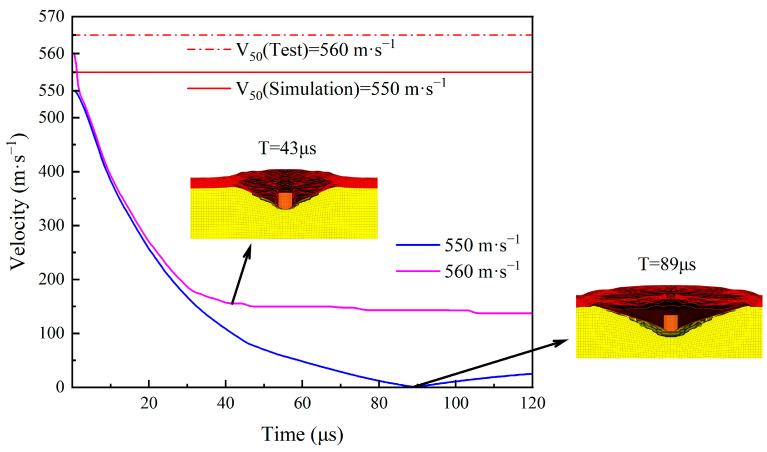
The velocity time history curve of the 1.1 g FSP.

Figure 2 shows the velocity-time curve of the 1.1 g FSP penetrating the UHMWPE target, where V_blocking_ = 550 m·s^−1^ and V_penetration_ = 560 m·s^−1^. From the velocity-time curves of the fragments in Figure 2, it can be seen that the fragment penetrated the body armor target plate at V = 560 m·s^−1^, while the fragment did not penetrate the target plate and eventually rebounded at V = 550 m·s^−1^. When the fragment’s speed is 550 m·s^−1^, its velocity rapidly decreases within the first 45 μs and then gradually decreases, reaching zero at t = 89 μs and starting to rebound. When the fragment’s speed is 560 m·s^−1^, its velocity rapidly decreases within the first 30 μs and then gradually decreases, penetrating the target plate at t = 43 μs and starting to enter the gelatin. Due to the effect of the gelatin, the fragment’s velocity exhibits a small stepwise decrease.

**Figure 3:**
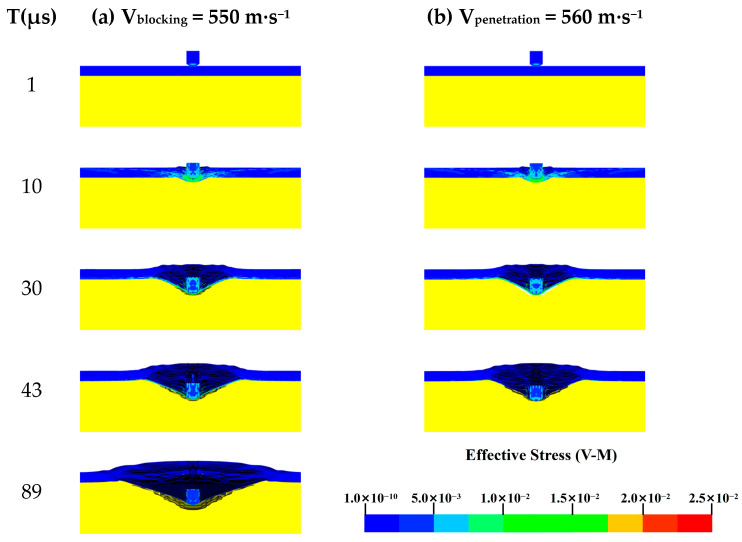
The Von Mises stress contour map of the FSP penetrating the body armor target plate: (**a**) V_blocking_ = 550 m·s^−1^; (**b**) V_penetration_ = 560 m·s^−1^.

The Von Mises stress contour map of the 1.1 g FSP impacting the UHMWPE body armor target plate at V_blocking_ = 550 m·s^−1^ and V_penetration_ = 560 m·s^−1^ are shown in Figure 3.

From Figure 3, the FSP did not penetrate the body armor target plate at a speed of 550 m·s^−1^, whereas penetration occurred at 43 μs when the projectile impacted at 560 m·s^−1^. The Von Mises stress contour map indicates that, in both scenarios, the failure of the body armor target plate is almost identical within the first 30 μs. At t = 1 μs, the FSP just begins to penetrate the body armor target plate, with only minor stresses appearing at the projectile’s tip and the impact site on the vest. By t = 10 μs, the body armor target plate experiences significant stress near the impact site due to the high-speed shearing action of the FSP, causing the initial layers of the target plate to fail rapidly and be penetrated. At t = 30 μs, the projectile’s speed decreases and its shearing capability diminishes, causing the unpenetrated layers of the vest to undergo tensile stress, accompanied by significant delamination. At t = 43 μs, the FSP with an initial velocity of 550 m·s^−1^ continues to penetrate the ballistic target, while the fragment with an initial velocity of 560 m·s^−1^ has already fully penetrated the target. At t = 89 μs, the initial velocity of the 550 m·s^−1^ projectile has reduced to zero, with only minor stresses remaining in the fragments and the last unpenetrated layer of the target plate.

**Figure 4:**
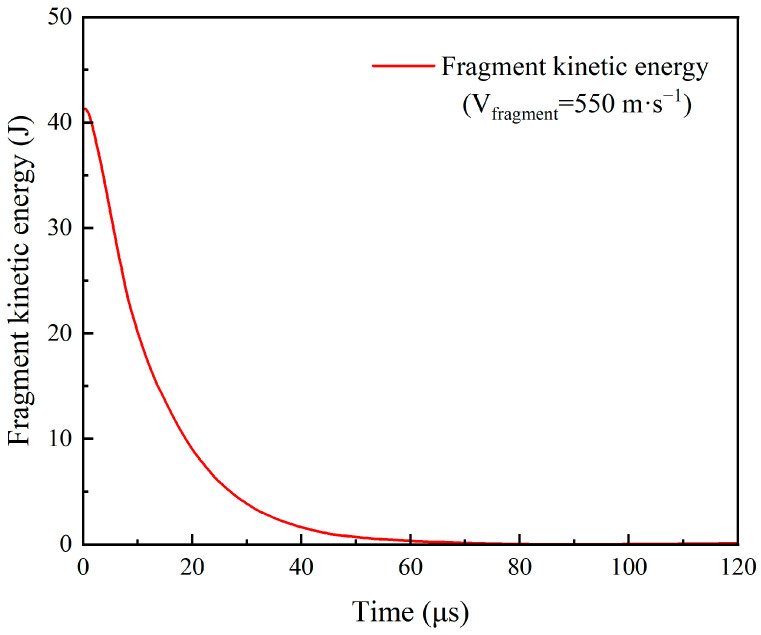
The trend in fragment kinetic energy variation over time.

This indicates that the high-speed penetration of the UHMWPE body armor target plate by the FSP involves three main processes: shearing failure, interlayer delamination, and tensile failure. From the trend in fragment kinetic energy over time at an initial velocity of 550 m/s shown in Figure 4, it can also be observed that during the early stage of penetration, which is the shear failure phase, the fragment consumes the most energy. In the later stages of penetration, during interlayer delamination and tensile failure, the energy loss is smaller until the kinetic energy is reduced to zero. Therefore, the shear failure phase is the primary failure mode during the target plate’s destruction process.

## 4. Results and Discussion

After validating the above numerical model, the material properties (elastic modulus, tensile strength, shear strength) and structural characteristics (number of layers, interlayer strength) of the UHMWPE body armor target plate were being studied based on the material properties and structural layout of the validated numerical model to understand their impact mechanisms on the ballistic limit velocity V_50_. Given the body armor’s thickness of only 4.124 mm, which is relatively small when being compared to the other two dimensions (X and Y), the deformation and failure in the thickness direction (Z direction) can be considered negligible. Therefore, when considering the impact of elastic modulus and tensile strength on the penetration resistance of UHMWPE ballistic plates, the elastic modulus and tensile strength in the Z direction (thickness direction) can be kept constant. This simplification allows the simulation process to focus on adjusting the elastic modulus and tensile strength in the X and Y directions, without accounting for variations in the Z direction and, thus, simplifies the simulation workflow.

### 4.1. The Impact of Material Properties on the V_50_ of the Body Armor Target Plate

#### 4.1.1. The Impact of Elastic Modulus on the V_50_ of the Body Armor Target Plate

Elastic modulus is an important parameter that reflects a material’s ability to resist deformation. The greater the elastic modulus, the stronger the material’s resistance to deformation and vice versa. Therefore, studying the impact mechanism of the elastic modulus on the penetration resistance of the UHMWPE body armor target plate has significant practical importance. Based on the experimentally obtained elastic modulus and referencing the elastic modulus of UHMWPE laminated plate available on the market, E_1_ = E_2_ = 30.70 GPa was set as the baseline with reasonable increase and decrease in the elastic modulus in X and Y directions. This approach helps understand the impact mechanism of changes in the elastic modulus in X and Y directions on the body armor’s resistance to penetration by a 1.1 g FSP. The simulation results are shown in Table 7.

**Figure 5:**
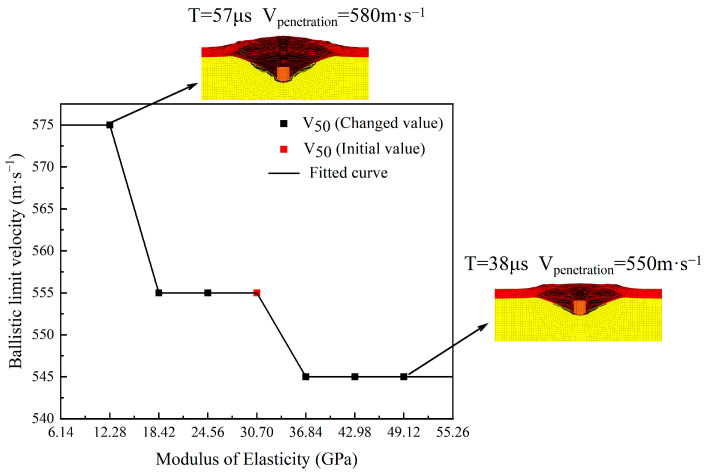
The trend in ballistic limit velocity with the variation of elastic modulus.

According to the results of the above numerical simulations, when the elastic modulus of the body armor target plate decreases from 30.70 GPa to 12.28 GPa, the ballistic limit velocity V_50_ increases from 555 m·s^−1^ to 575 m·s^−1^, an increase of 4%. When the elastic modulus increases from 30.70 GPa to 36.84 GPa, the ballistic limit velocity V_50_ decreases to 545 m·s^−1^, a reduction of 2%. This is attributable to the fact that as the elastic modulus of the bulletproof target plate increases, it becomes harder and more challenging to deform. Consequently, penetration occurs before the material can reach its ultimate tensile deformation. In contrast, when the elastic modulus decreases, the body armor target plate becomes softer and can produce greater elastic deformation, making it relatively harder to penetrate. The rate of change in the ballistic limit velocity V_50_ with changes in the elastic modulus ranges from −2% to 4%. Additionally, the trend in the ballistic limit velocity V_50_ with changes in the elastic modulus is shown in Figure 5.

According to the trend shown in Figure 5, as the elastic modulus of the body armor target plate increases, the ballistic limit velocity V_50_ tends to decrease. This is like the conclusion reached by Heisserer [38]: with constant strength, increasing the modulus leads to a decrease in the ballistic limit velocity V_50_, whereas decreasing the elastic modulus results in an increase in the ballistic limit velocity V_50_. Figure 5 also shows that when the elastic modulus of the body armor target plate is between 18.42 and 30.70 GPa, the ballistic limit velocity V_50_ remains unchanged. When the elastic modulus continues to decrease below 18.42 GPa, the ballistic limit velocity V_50_ exhibits a stepwise increase, rising from 555 m·s^−1^ to 575 m·s^−1^. In contrast, when the elastic modulus increases above 30.70 GPa, the ballistic limit velocity V_50_ shows a stepwise decrease, dropping from 555 m·s^−1^ to 545 m·s^−1^. By analyzing the failure modes of the body armor target plate at elastic moduli of 12.28 GPa and 49.12 GPa, it can be observed that in both cases, the target plate exhibited significant delamination. However, with a lower elastic modulus, the target plate exhibited a more pronounced tensile deformation capability, allowing its tensile strength to be more fully utilized. Based on this, it can be inferred that materials with a lower elastic modulus might perform better in resisting fragment penetration.

In summary, the change in ballistic limit velocity V_50_ with elastic modulus is not continuous but more like the segmented changes shown in the fitted function curve in the figure. The ballistic limit velocity V_50_ only changes accordingly when the elastic modulus increases or decreases to a certain critical value. The above results indicate that the influence of the elastic modulus on the ballistic limit velocity V_50_ is minimal.

#### 4.1.2. The Impact of Tensile Strength on the V_50_ of the Body Armor Target Plate

Tensile strength is an important parameter for a material’s resistance to tensile failure, especially in the context of high-speed fragment penetration. Excellent tensile strength can play a crucial role in the later stages of penetration. The UHMWPE body armor target plate undergoes three stages during fragment penetration: shear failure, shear plugging, and tensile failure. In the later stages of high-speed fragment penetration, the fragment’s speed decreases, reducing its shear damaging capability on the target plate. At this stage, the UHMWPE layer that has not yet been penetrated is primarily subjected to tensile forces. Strong tensile strength can effectively stop the fragments outside the human body, preventing further injury. Based on the existing tensile strength Xt = Yt = 3.10 GPa of the UHMWPE body armor target plate, a reasonable gradient variation by using numerical simulation to study the impact of changes in tensile strength on the ballistic limit V_50_ of the target plate was made in this paper, and the results are listed in Table 8.

From the ballistic limit velocity V_50_ results of the 1.1 g FSP penetrating the UHMWPE body armor target plate under the above seven sets of tensile strength variations, it can be seen that when the tensile strength decreases from 3.10 GPa to 1.24 GPa, the ballistic limit velocity V_50_ of the body armor target plate decreases significantly from 555 m·s^−1^ to 385 m·s^−1^, a reduction of 31%. In contrast, when the tensile strength increases from 3.10 GPa to 4.34 GPa, the ballistic limit velocity V_50_ increases from 555 m·s^−1^ to 565 m·s^−1^, an increase of 2%. This indicates that the ballistic limit velocity V_50_ of the body armor target plate is influenced by its tensile strength. When the tensile strength decreases, the ballistic limit velocity V_50_ also decreases. However, when the tensile strength increases, the change in the ballistic limit velocity V_50_ is not significant, with only a slight increase. Further increasing the tensile strength reveals that the ballistic limit velocity V_50_ of the body armor target plate does not continue to increase accordingly.

**Figure 6:**
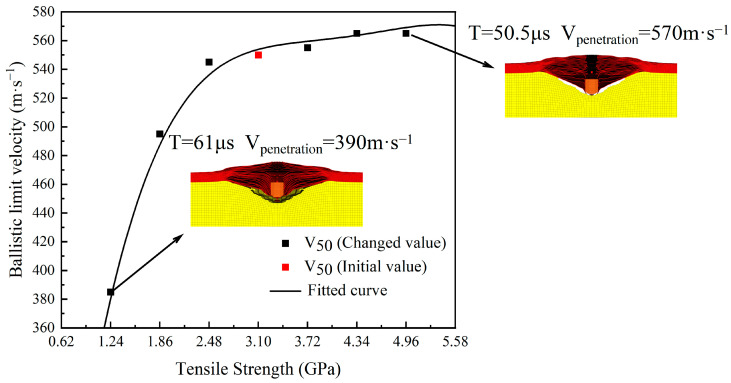
The trend in ballistic limit velocity with the variation of tensile strength.

From the trend in ballistic limit velocity of the body armor target plate with tensile strength shown in Figure 6, the ballistic limit velocity V_50_ of the body armor target plate generally shows an upward trend with the increase in tensile strength. When the tensile strength of the body armor target plate is less than 2.48 GPa, the slope of the fitted function curve increases rapidly, and its ballistic limit velocity V_50_ begins to decrease significantly, indicating a substantial reduction in the penetration resistance of the body armor target plate. When the tensile strength of the body armor target plate exceeds 2.48 GPa, the slope of the fitted function curve gradually levels off, and further increases in tensile strength have an insignificant impact on the ballistic limit velocity V_50_, resulting in only slight increases. By analyzing the failure modes at tensile strengths of 1.24 GPa and 4.96 GPa, it can be observed that the increase in tensile strength is accompanied by an increase in tensile deformation. This trend shows that as tensile strength increases, the material’s ability to resist tensile failure also correspondingly improves. In the later stages of fragment penetration of the body armor target plate, excellent tensile performance becomes particularly important, as the failure mode of the body armor target plate at this stage primarily manifests as tensile failure. Therefore, the higher the tensile strength, the greater the tensile deformation the material can withstand until the tensile deformation reaches the critical point of failure.

Therefore, when the tensile strength of the body armor target plate significantly decreases, the ballistic limit velocity is very sensitive to changes in tensile strength. At this stage, the body armor target plate primarily fails due to tensile failure. In contrast, when the tensile strength of the body armor target plate increases, the change in ballistic limit velocity V_50_ is not significant. The reason for this is that at this stage, the primary failure mode of the body armor target plate is shear failure. Further increasing the tensile strength of the body armor target plate has minor impact on its penetration resistance, because the target plate has already experienced shear failure before reaching its tensile limit, which also explains the results obtained in Table 8.

#### 4.1.3. The Impact of Shear Strength on the V_50_ of the Body Armor Target Plate

Shear strength refers to the ability of a material to resist shear failure. The greater the shear strength, the stronger its ability to resist shear failure and vice versa. Excellent shear strength plays a crucial role in the field of high-speed fragment penetration. When an FSP penetrates a body armor target plate, the high initial velocity of the fragment results in an extraordinarily strong shear capability. At this moment, the excellent shear performance of the body armor target plate will significantly dissipate the kinetic energy of the FSP, greatly reducing its speed. Therefore, shear strength is critical to the ballistic limit velocity V_50_ of the body armor target plate. The impact of changes in shear strength on ballistic limit velocity V_50_ is shown in Table 9.

From the above seven sets of data, it can be concluded that when the shear strength decreases from 0.55 GPa to 0.22 GPa, the ballistic limit velocity V_50_ of the body armor target plate decreases from 550 m·s^−1^ to 455 m·s^−1^, a decrease of 18%. When the shear strength increases from 0.55 GPa to 0.88 GPa, the ballistic limit velocity V_50_ increases from 555 m·s^−1^ to 615 m·s^−1^, an increase of 11%. The influence of shear strength on the ballistic limit velocity V_50_ of the body armor target plate is quite significant. The ballistic limit velocity V_50_ changes with the shear strength; when the shear strength decreases, the ballistic limit velocity V_50_ also decreases and vice versa.

**Figure 7:**
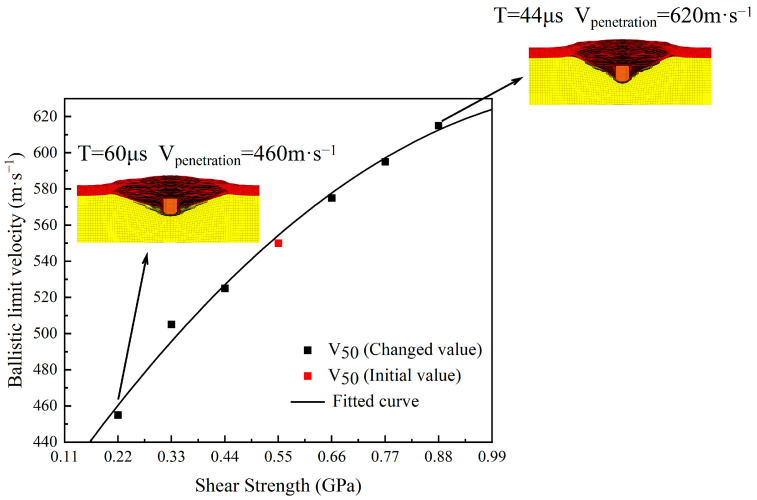
The trend in ballistic limit velocity with the variation of shear strength.

From the trend in the ballistic limit velocity V_50_ with shear strength shown in Figure 7, it can be seen that as the shear strength of the body armor target plate increases, its ballistic limit velocity V_50_ generally shows an upward trend, which is exactly the opposite of the results obtained by Karthikeyan et al. [45]. The reason may be that the UHMWPE plate studied by Karthikeyan et al. was relatively thick, whereas, for a thinner plate, shear strength becomes the main cause of failure. Additionally, the inconsistency in results might also be influenced by the matrix material. As can also be seen from the failure morphology of the body armor target plate at shear strengths of 0.22 GPa and 0.88 GPa in Figure 7, the tensile deformation of the body armor target plate at a shear strength of 0.88 GPa is slightly greater than at 0.22 GPa. This indirectly proves that excellent shear strength is a sufficient condition for the tensile strength to take effect.

Unlike tensile strength, the ballistic limit velocity of the body armor target plate increases with the rise in shear strength. This is because shear failure is the first form of failure during the fragment’s penetration of the target plate, and at this stage, the fragment consumes the most energy. Therefore, as shear strength increases, the penetration resistance of the body armor target plate also increases, which is unlike tensile strength maintaining a constant effect after reaching a certain level. Hence, the excellent shear strength of the body armor target plate is crucial for its penetration resistance performance.

In summary, the elastic modulus, tensile strength, and shear strength of the target plate have varying degrees of influence on its ballistic limit velocity. Among them, shear strength has the most significant impact, followed by tensile strength, with the elastic modulus having the least effect. The reason is that the elastic modulus primarily affects the deformation capacity of the target plate, and its influence on resistance to failure is not as pronounced. In contrast, shear strength and tensile strength represent the target plate’s ability to resist shear failure and tensile failure, respectively. Changes in these strengths have a more significant impact on the plate’s resistance to damage. Additionally, as mentioned earlier, the shear failure phase is the stage where the most energy is consumed during fragment penetration. Therefore, increasing the shear strength of the target plate material significantly enhances its ballistic performance.

### 4.2. The Impact of Structural Characteristics on the V_50_ of the Body Armor Target Plate

The structural characteristics of body armors are equally important to their ballistic limit velocity V_50_. With carefully designed structural layouts, body armors can further improve their penetration resistance while maintaining lightness and comfort. Therefore, this section reasonably varies the number of layers and the interlayer bonding strength of the body armor target plate to study the impact mechanism of these structural characteristics on its ballistic limit velocity V_50_.

#### 4.2.1. The Impact of the Number of Layers on the V_50_ of the Body Armor Target Plate

When a fragment penetrates a ballistic target, it undergoes three stages: shear failure, interlayer delamination, and tensile failure. Interlayer delamination can dissipate part of the energy from the high-velocity fragment. Thus, from a macroscopic perspective, changes in the number of layers will affect the overall interlayer delamination behavior. The more layers there are, the more energy is consumed by interlayer delamination. From a microscopic perspective, changes in the number of layers affect the thickness of each individual layer, which indirectly impacts the overall structure’s ballistic limit velocity. The more layers there are, the thinner each layer becomes, which reduces the penetration resistance of each layer. Therefore, studying the effect of the number of layers on the ballistic limit velocity when the target plate thickness is constant holds important theoretical and practical significance. Based on the original body armor target plate model (23 layers, total thickness 4.124 mm), several other finite element models of body armor target plates with the same thickness but different numbers of layers were established. Finite element simulations were performed on each of these models, and the ballistic limit velocity V_50_ results for body armor target plates with the same thickness but different numbers of layers were obtained and listed in Table 10.

According to the results in Table 10, when the number of layers is reduced from 23 to 13 or 18, the ballistic limit velocity V_50_ does not change. However, when the number of layers is further reduced to 8, the ballistic limit velocity V_50_ increases significantly from 555 m·s^−1^ to 605 m·s^−1^, with an increase of 9%. On the other hand, when the number of layers is increased from 23 to 28, the ballistic limit velocity V_50_ decreases from 555 m·s^−1^ to 545 m·s^−1^, with a decrease of 2%. When the number of layers continues to increase, the ballistic limit velocity V_50_ dose no longer changes. This indicates that increasing the number of layers has a very minimal impact on the ballistic limit velocity V_50_, while appropriately reducing the number of layers can improve the ballistic limit velocity V_50_.

**Figure 8:**
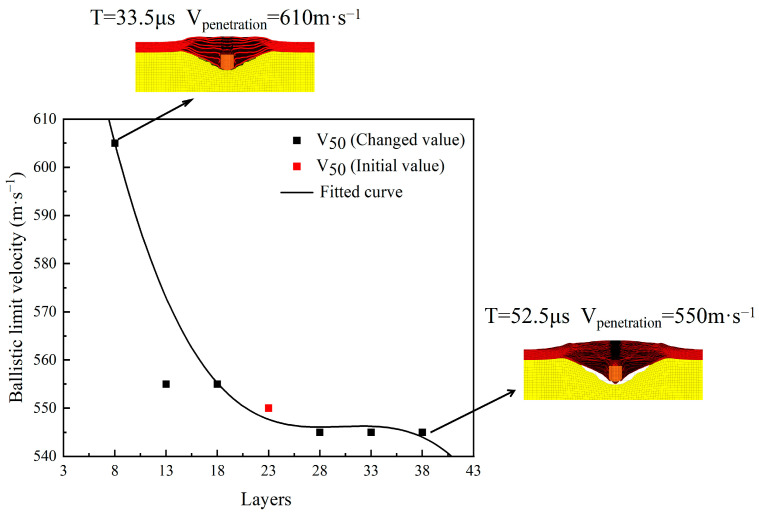
The trend in ballistic limit velocity with the variation of the number of layers.

From the trend in the ballistic limit velocity V_50_ of the body armor target plates with respect to the number of layers in Figure 8, it can be seen that when the total thickness of the body armor target plate remains unchanged, the ballistic limit velocity V_50_ generally shows a decreasing trend as the number of layers increases. The fitted function curve shows that when the number of layers in the body armor target plate is above 13, the change in ballistic limit velocity is not significant. However, when the number of layers decreases to below 13, the ballistic limit velocity V_50_ increases significantly.

By comparing differences in damage patterns between body armor target plates of 8 layers and 38 layers, it can be observed that tensile deformation is not significant in both cases, indicating that shear failure is the dominant failure mechanism in these situations. With the total thickness kept constant, as the number of layers increases, the thickness of each layer decreases correspondingly, leading to a significant reduction in the shear resistance of each layer. This structural weakness allows high-speed fragments to penetrate each layer of the target plate relatively easily. In contrast, when the number of layers in the body armor target plate is significantly reduced, the thickness of each layer increases correspondingly, thereby enhancing the shear resistance of each layer. This enhanced shear performance effectively slows down the velocity of the fragments in the initial penetration stage, reducing their ability to penetrate subsequent layers. Therefore, the ballistic limit velocity V_50_ of body armor target plates significantly increases when the number of layers is reduced.

In summary, when the total thickness remains constant, a smaller number of layers can improve the shear resistance of the structure, thereby enhancing the overall protective performance of the body armor target plate, leading to an increase in the ballistic limit velocity V_50_. This conclusion is slightly different from that of Zhang et al. [46], possibly because the total thickness of the laminated plates in their study was much greater than the thickness of the body armor target plates provided in this study. When the number of layers increases, the thickness of each target plate layer is not excessively small, so the shear resistance of each layer remains relatively high, leading to different results. Additionally, their study considered the cross-laminated structure of UHMWPE, which might be another reason explaining the difference. Therefore, when the total thickness of the body armor target plate is relatively thin, having too many layers can reduce its penetration resistance.

#### 4.2.2. The Impact of Interlayer Strength on the V_50_ of the Body Armor Target Plate

From the failure modes of the target plate, it is evident that interlayer delamination is one of the key failure mechanisms during the penetration of the target by a fragment. Interlayer strength significantly affects this delamination, making the study of its influence on the ballistic limit velocity of the target plate of great theoretical significance. The above studies examined the effects of the material properties and the number of layers of body armor target plates on their ballistic limit velocity V_50_. When considering the above factors, the bonding between the layers of the body armor target plates was not taken into account, so the contact between each layer of the target plates was set to *CONTACT_AUTOMATIC_SURFACE_TO_SURFACE. This section studies the effect of interlayer strength of body armor target plates on their ballistic limit velocity V_50_. Therefore, the contact between each layer of the target plates was changed to *CONTACT_AUTOMATIC_SURFACE_TO_SURFACE_TIEBREAK, which allowed for the failure of the bonded interface between the two contacting surfaces during the contact process. The interlayer failure criterion is shown in Equation (2), where NFLS and SFLS represent the interlayer normal strength and the interlayer shear strength, respectively [28] and σ_n_ and σ_s_ represent the normal failure stress and shear failure stress of the adhesive layer, respectively. In this study, the interlayer normal strength (NFLS) is 55 MPa, and the interlayer shear strength (SFLS) amounts to 120 MPa [47]. Based on the above interlayer failure strength, the impact of changes in interlayer strength on the ballistic limit velocity V_50_ of the body armor target plates was studied.

(2) $${\left(\frac{\left|\sigma_{n}\right|}{NFLS}\right)}^{2}+{\left(\frac{\left|\sigma_{s}\right|}{SFLS}\right)}^{2}\geq 1$$

**Figure 9:**
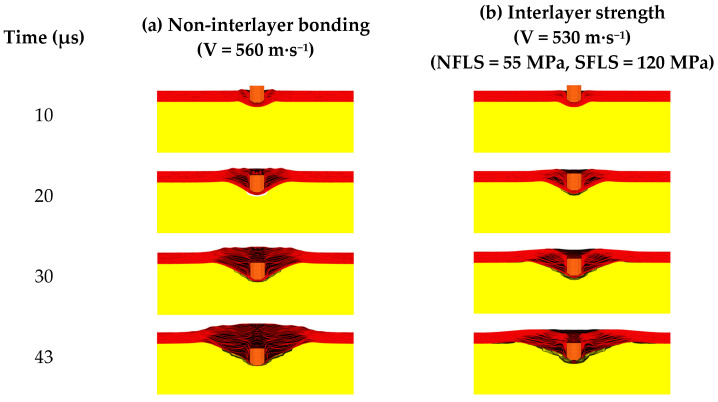
The failure process of the body armor target plate: (**a**) non-interlayer strength; (**b**) interlayer strength (NFLS = 55 MPa, SFLS = 120 MPa).

When the aforementioned interlayer strength values are added to the finite element model, the ballistic limit velocity V_50_ of the body armor target plate decreases from 555 m·s^−1^ to 525 m·s^−1^, a reduction of 5%. Additionally, the damage process diagrams of the body armor target plates in both cases (without interlayer bonding and with the interlayer strength) are presented in Figure 9.

From Figure 9, the damage process of body armor target plates with and without interlayer strength shows that the presence or absence of interlayer bonding has little effect on the shear failure of the body armor target plates, with both exhibiting similar damage forms within the first 20 μs. When the fragments continue to penetrate and reach 30 μs, delamination of the body armor target plates begins to occur. At this point, the body armor target plates without interlayer bonding exhibit more pronounced delamination and slightly greater back deformation compared to the body armor target plates with interlayer strength (NFLS = 55 MPa, SFLS = 120 MPa). At 43 μs, both types of plates are penetrated, with the delamination and back deformation of the non-bonded body armor target plates being significantly greater than those with the interlayer strength (NFLS = 55 MPa, SFLS = 120 MPa). In the later stages of fragment penetration, the non-bonded body armor target plates exhibit more significant delamination and greater tensile deformation. Therefore, the ballistic limit velocity V_50_ of the non-bonded body armor target plates is higher than that of the body armor target plates with the above interlayer strength (NFLS = 55 MPa, SFLS = 120 MPa).

Based on the above results, interlayer strength has a certain impact on the ballistic limit velocity V_50_ of body armor target plates. Therefore, based on the interlayer strengths listed in Table 11, the interlayer normal strength and interlayer shear strength are proportionally varied to explore the impact of interlayer strength changes on the ballistic limit velocity V_50_ of body armor target plates.

First, the interlayer normal strength is proportionally varied while keeping the interlayer shear strength constant, and the results obtained from numerical simulations are listed in Table 11.

From Table 11, it can be seen that when the interlayer normal strength of the body armor target plate is 0.55 MPa and the interlayer shear strength is 120 MPa, the ballistic limit velocity V_50_ of the body armor target plate decreases from 555 m·s^−1^ to 545 m·s^−1^, a reduction of 2%. When the interlayer normal strength is 5500 MPa and the interlayer shear strength is 120 MPa, the ballistic limit velocity V_50_ decreases from 555 m·s^−1^ to 525 m·s^−1^, a reduction of 5%.

**Figure 10:**
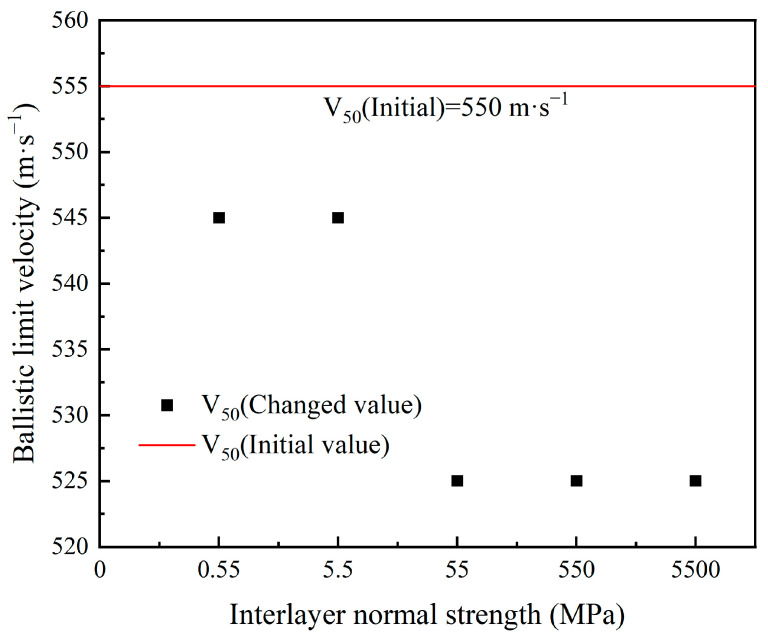
The trend in ballistic limit velocity with the variation of interlayer normal strength.

From Figure 10, it can be observed that the ballistic limit velocity V_50_ of the body armor target plate decreases gradually with the increase in interlayer normal strength. In addition, the ballistic limit velocity V_50_ of the body armor target plates with the interlayer strength values listed in Table 11 is lower than that of the body armor target plates without interlayer bonding.

**Figure 11:**
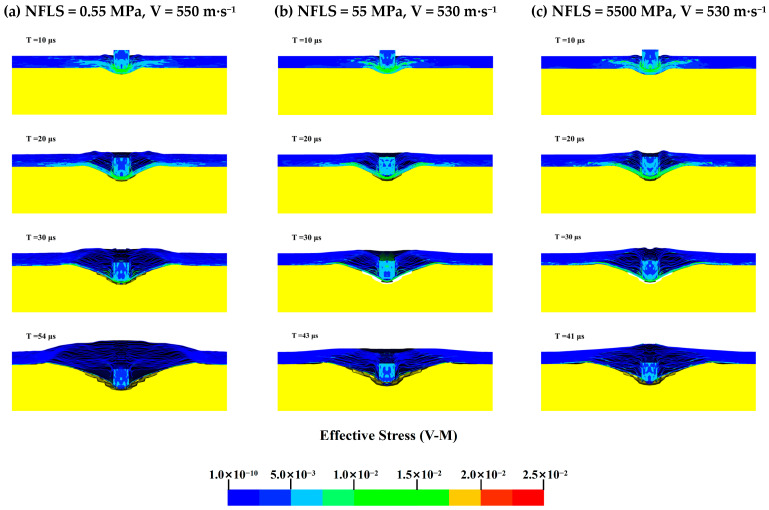
The Von Mises stress contour map of the body armor target plate: (**a**) NFLS = 0.55 MPa, V = 550 m·s^−1^; (**b**) NFLS = 55 MPa, V = 530 m·s^−1^; (**c**) NFLS = 5500 MPa, V = 530 m·s^−1^.

To further determine the effect of the interlayer normal strength on the ballistic limit velocity V_50_ of the body armor target plate, the Von Mises stress cloud diagrams for NFLS values of 0.55, 55, and 5500 MPa when SFLS is 120 MPa are shown in Figure 11. The penetration process is analyzed accordingly.

From the Von Mises stress cloud diagrams in Figure 11 for NFLS values of 0.55, 55, and 5500 MPa, it can be seen that during the initial 20 μs, the failure modes of the body armor target plates for the three interlayer strengths are essentially the same, all exhibiting shear failure with similar stress levels. When penetration continues up to 30 μs, the delamination and back deformation of the target plates differ due to the varying interlayer normal strengths. At an NFLS of 0.55 MPa, the delamination and back deformation of the body armor target plate are significantly greater than at NFLS values of 55 MPa and 5500 MPa. For NFLS values of 55 MPa and 5500 MPa, the failure modes of the body armor target plates are the same at large. Thus, it can be concluded that once the interlayer normal strength of the body armor target plate increases beyond a certain range, further increases in interlayer normal strength have a minimal impact on the ballistic limit velocity V_50_.

After analyzing the effect of interlayer normal strength on the ballistic limit velocity V_50_ of the body armor target plate, the focus shifts to studying the effect of interlayer shear strength on the ballistic limit velocity V_50_ of the body armor target plate. By keeping the interlayer normal strength constant (NFLS = 55 MPa) and proportionally varying the interlayer shear strength, the influence of changes in interlayer shear strength on the ballistic limit velocity V_50_ of the body armor target plate is studied. The obtained results are presented in Table 12.

From Table 12, when the interlayer shear strength is 1.2 MPa and the interlayer normal strength is 55 MPa, the ballistic limit velocity V_50_ of the body armor target plate increases from 555 m·s^−1^ to 575 m·s^−1^, with an increase of 4%. In contrast, when the interlayer shear strength is 12,000 MPa and the interlayer normal strength is 55 MPa, the ballistic limit velocity V_50_ decreases from 555 m·s^−1^ to 445 m·s^−1^, with a decrease of 20%.

**Figure 12:**
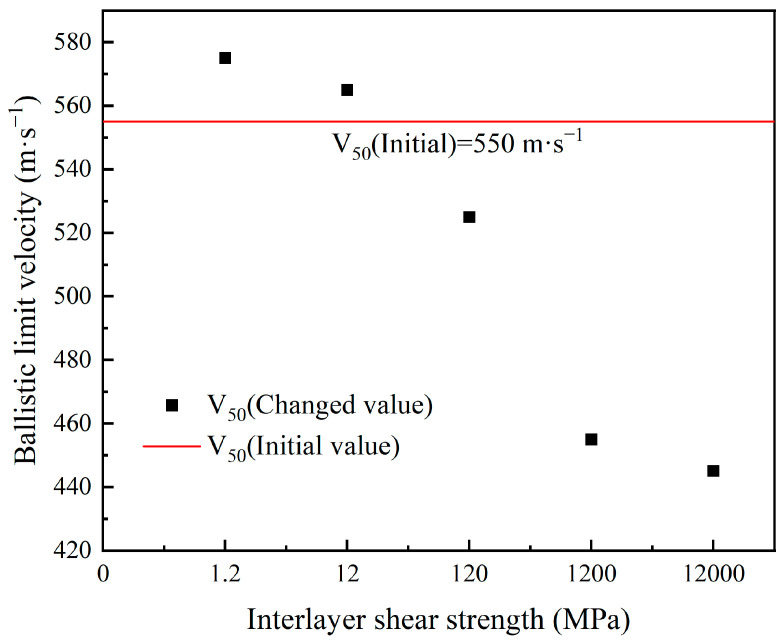
The trend in ballistic limit velocity with the variation of interlayer shear strength.

From the trend in Figure 12 showing the ballistic limit velocity with changes in interlayer shear strength, when the interlayer shear strength of the body armor target plate is 1.2 or 12 MPa, its ballistic limit velocity V_50_ is greater than that of the target plate without interlayer bonding. However, as the interlayer shear strength continues to increase, the ballistic limit velocity V_50_ becomes lower than that of the target plate without interlayer bonding. It can be seen that the ballistic limit velocity V50 of the body armor target plate first increases and then decreases with the increase in interlayer shear strength.

**Figure 13:**
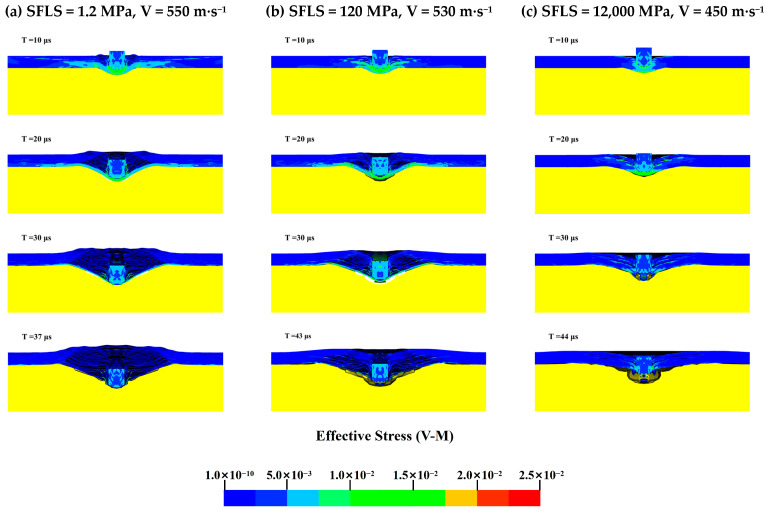
The Von Mises stress contour map of the body armor target plate: (**a**) SFLS = 1.2 MPa, V = 550 m·s^−1^; (**b**) SFLS = 120 MPa, V = 530 m·s^−1^; (**c**) SFLS = 12,000 MPa, V = 450 m·s^−1^.

To further determine the effect of interlayer shear strength on the ballistic limit velocity V_50_ of the body armor target plate, the Von Mises stress cloud diagrams for SFLS values of 1.2, 120, and 12,000 MPa when NFLS is 55 MPa are shown in Figure 13. The penetration process is analyzed accordingly.

From the Von Mises stress cloud diagrams in Figure 13 for SFLS values of 1.2, 120, and 12,000 MPa, as the interlayer shear strength of the body armor target plate increases, delamination and tensile deformation significantly decrease. At an SFLS of 1.2 MPa, the body armor target plate primarily experiences shear failure in the first 20 μs, significant tensile deformation at 30 μs, and penetration by 37 μs. When SFLS is 120 MPa, the failure mode of the body armor target plate is essentially the same as at 1.2 MPa, but both delamination and tensile deformation are less than at 1.2 MPa. At an SFLS of 12,000 MPa, the failure mode of the body armor target plate is completely different from the previous two; the plate mainly undergoes shear failure and shear plugging with almost no interlayer delamination or tensile deformation. At this point, the failure mode of the body armor target plate is like that of a single-layer plate of the same thickness.

In summary, the interlayer strength of the body armor target plate has a significant impact on its ballistic performance, with the effect of interlayer shear strength being much greater than that of interlayer normal strength. When the body armor target plate has appropriate interlayer strength, the delamination between the layers can also dissipate part of the projectile’s kinetic energy, making it more difficult to penetrate the target plate. However, if the interlayer strength is too high, the failure behavior of the body armor target plate is similar to that of a single-layer plate of the same thickness, and its failure mode is entirely different from that of a multi-layered structure. In this case, the primary causes of failure are shear failure and shear plugging, with almost no delamination or tensile deformation occurring [48]. Therefore, determining the appropriate interlayer strength between the layers of the body armor target plate is crucial for enhancing its ballistic performance.

## 5. Conclusions

This article conducts a study on the penetration of 1.1 g FSP into a UHMWPE target plate and reaches the following conclusions:

(1) Based on the experimental results, a numerical model of a 1.1 g FSP penetrating a UHMWPE target was established, and the numerical simulation results closely match the experimental data. Additionally, a simulation of the ballistic limit velocity of the target was conducted, revealing that the failure process of the target primarily involves three failure modes: shear failure, interlayer delamination, and tensile failure.
(2) The material parameters of the ballistic target significantly influence its ballistic limit velocity. A sensitivity analysis was conducted to assess how the elastic modulus, tensile strength, and shear strength affect the ballistic limit velocity. The results indicate that the sensitivity of the ballistic limit velocity to material parameters follows this order, shear strength has the most significant impact, followed by tensile strength, with the elastic modulus having the least effect.
(3) The structural characteristics of the ballistic target also have a significant impact on its ballistic limit velocity. The effect of varying the number of layers in targets of the same thickness on the ballistic limit velocity was studied, and the results show that as the number of layers increases, the ballistic limit velocity decreases. In addition, the effect of interlayer strength on the ballistic limit velocity was also investigated. As the interlayer strength increases, the ballistic limit velocity initially increases but then decreases.

## Figures and Tables

**Table 1:** Common ballistic fiber material parameters.

Fiber Properties	Fiber Density (kg/m^3^)	Modulus (GPa)	Strength (GPa)	Failure Strain (%)	Tenacity (N/tex)	Wave Speed (km/s)
UHMWPE (Dyneema^®^ SK76)	970	116	3.6	3.8	3.7	10.9
Aramid (Kevlar^®^ KM2)	1440	83	3.4	3.6	2.4	7.6
Carbon	1800	227	3.8	1.8	2.1	11.2
E-Glass	2550	74	3.5	4.7	1.4	5.4

**Table 2:** The effect of element size on numerical results.

Element Size/(mm)	V_50_		Error/%	Calculation Time/h
	(Numerical Simulation)/m·s^−1^	(Experimental Results)/m·s^−1^		
0.2	557	565	−1.4	7.8
0.4	555	565	−1.8	4.3
0.6	544	565	−3.7	3.1

**Table 3:** The material parameters of UHMWPE body armor target plate.

ρ/(g·cm^−3^)	E_1_/Gpa	E_2_/Gpa	E_3_/Gpa	μ_12_	μ_13_	μ_23_	G_12_/Gpa	G_13_/Gpa	G_23_/Gpa
0.97	30.70	30.70	1.97	0.008	0.044	0.044	0.73	0.67	0.67
**S_BA_/Gpa**	**S_CA_/Gpa**	**S_CB_/Gpa**	**X_t_/Gpa**	**Y_t_/Gpa**	**Z_t_/Gpa**	**Y_c_/Gpa**			
0.55	0.55	0.55	3.10	3.10	0.95	1.30			

**Table 4:** The material parameters of 1.1 g FSP.

ρ/(kg·m^−3^)	*E*/Gpa	${\dot{\varepsilon }}_{0}$/s^−1^	*v*	*A*/Mpa	*B*/Mpa	*n*	*C*	*m*
7800	300	1	0.3	507	320	0.28	0.064	1.06
***C*** **_P_** **/J·kg^−1^·K^−1^**	***T*** **_r_** **/K**	***T*** **_m_** **/K**	***D*** **_1_**	***D*** **_2_**	***D*** **_3_**	***D*** **_4_**	***D*** **_5_**	
469	300	1795	0.10	0.76	1.57	0.005	−0.84	

**Table 5:** The material parameters of Gelatin.

*ρ*/(g·cm^−3^)	*G*/Gpa	*SIGY*/Gpa	*EH*/Gpa	*C*_0_/Gpa	*C*_1_/Gpa	*C*_2_/Gpa	*C*_3_/Gpa
1.03	2.63 × 10^−7^	1.17 × 10^−7^	1.63 × 10^−8^	0	2.38	7.14	11.9

**Table 6:** Comparison between experimental and numerical simulation results.

1.1 g FSP Penetration of UHMWPE Body Armor	V_50_ (m·s^−1^)	Error (%)
Experimental results	565	−1.8
Numerical simulation	555	

**Table 7:** The impact of elastic modulus on the V_50_ of body armor target plate.

Elastic Modulus (E_1_ = E_2_)/(GPa)	V_blocking_/(m·s^−1^)	V_penetration_/(m·s^−1^)	V_50_/(m·s^−1^)	Rate of Change(%)
12.28	570	580	575	+4
18.42	550	560	555	0
24.56	550	560	555	0
30.70	550	560	555	/
36.84	540	550	545	−2
42.98	540	550	545	−2
49.12	540	550	545	−2

**Table 8:** The impact of tensile strength on the V_50_ of body armor target plate.

Tensile Strength (X_t_ = Y_t_)/(GPa)	V_blocking_/(m·s^−1^)	V_penetration_/(m·s^−1^)	V_50_/(m·s^−1^)	Rate of Change (%)
1.24	380	390	385	−31
1.86	490	500	495	−11
2.48	540	550	545	−2
3.10	550	560	555	/
3.72	550	560	555	0
4.34	560	570	565	+2
4.96	560	570	565	+2

**Table 9:** The impact of shear strength on the V_50_ of body armor target plate.

Shear Strength (S_ba_= S_ca_ = S_cb_)/(GPa)	V_blocking_/(m·s^−1^)	V_penetration_/(m·s^−1^)	V_50_/(m·s^−1^)	Rate of Change (%)
0.22	450	460	455	−18
0.33	500	510	505	−9
0.44	520	530	525	−5
0.55	550	560	555	/
0.66	570	580	575	+4
0.77	590	600	595	+7
0.88	610	620	615	+11

**Table 10:** The impact of the number of layers on the V_50_ of body armor target plate.

Number of Layers	V_blocking_/(m·s^−1^)	V_penetration_/(m·s^−1^)	V_50_/(m·s^−1^)	Rate of Change (%)
8	600	610	605	+9
13	550	560	555	0
18	550	560	555	0
23	550	560	555	/
28	540	550	545	−2

**Table 11:** The impact of interlayer normal strength on the V_50_ of body armor target plate.

NFLS/(MPa)	SFLS/(MPa)	V_50_/(m·s^−1^)	Rate of Change (%)
0.55	120	545	−2
5.50	120	545	−2
55.00	120	525	−5
550.00	120	525	−5
5500.00	120	525	−5

**Table 12:** The impact of interlayer shear strength on the V_50_ of body armor target plate.

SFLS/(MPa)	NFLS/(MPa)	V_50_/(m·s^−1^)	Rate of Change (%)
1.2	55	575	+4
12.0	55	565	+2
120.0	55	525	−5
1200.0	55	455	−18
12,000.0	55	445	−20

## Data Availability

Data are contained within the article.
